# Chronic Lymphocytic Leukemia (CLL)-Derived Extracellular Vesicles (EVs) Modulate Monocytes to Become CLL-Supportive Cells

**DOI:** 10.3390/ijms27104638

**Published:** 2026-05-21

**Authors:** Shaked Noah, Einat Be’ery, Zinab Sarsor, Aladin Samara, Pia Raanani, Orit Uziel

**Affiliations:** 1 The Felsenstein Medical Research Center, Rabin Medical Center, Petah Tikva 4941492, Israel; shakednoah91@gmail.com (S.N.); einat.beery@gmail.com (E.B.); zeinabsa1@clalit.org.il (Z.S.); aladin.samara@gmail.com (A.S.); 2 Institute of Hematology, Davidoff Cancer Center, Petah Tikva 4941492, Israel; 3 The Gray Faculty of Medical & Health Sciences, Tel-Aviv University, Ramat-Aviv, Tel Aviv 6139001, Israel

**Keywords:** exosomes, CLL, monocytes, fibrocytes, cell–cell communication, survival advantage

## Abstract

In light of our previous publication, we hypothesized that chronic lymphocytic leukemia (CLL) cells also recruit monocytes to acquire survival advantage. To test this, we treated Buffy coat-driven monocytes with exosomes isolated from the peripheral blood of 45 treatment-naïve patients. The CLL-derived exosomes turned monocytes into IL-6-producing cells as an increase of 13-fold in the IL-6 levels was obtained in the growth medium of the exposed monocytes. Subsequently, we filtered out the monocytes and added CLL cells to this IL-6 enriched medium. As a result, the oncogene STAT3 became phosphorylated, and thus may have provided the cells with a survival advantage. A total of 67 phosphoproteins were upregulated in response to CLL-derived exosomal exposure in the recipient monocytes, with TFIIF being among the top scored proteins in this analysis. Transfection of monocytes with a TFIIF-containing vector increased the levels of IL-6 about 14-fold in the culture medium. Importantly, the CLL-derived exosomes induced the transformation of a portion of the recipient monocytes (45% compared to 30% of the unexposed cells) to become nurse-like fibrocyte cells. Taken together, CLL cells communicate with monocytes through the exosomes that they release. Once they are taken up by monocytes, they turn them into IL-6-producing cells, which provide a survival advantage to the neoplastic cells, creating a vicious circle that promotes disease progression.

## 1. Introduction

Chronic lymphocytic leukemia (CLL) is a dynamic blood cell disease which currently has no complete cure [1,2,3,4]. Under laboratory conditions, CLL cells undergo apoptosis within 7–14 days of cell division, unless they grow in the presence of stem cells or monocytes that inhibit this programmed cell death [3,5]. As such, CLL cells survive and actually depend (among others) on their interactions with the surrounding cells, including monocytes [6]. However, a comprehensive mechanism by which monocytes support the growth of CLL cells has not been fully elucidated.

Exosomes are small vesicles (30–150 nm) that are secreted from all cells in the body into various body fluids. They are surrounded by a bilayer membrane containing a molecular cargo which is mostly similar to their cells of origin, including nucleic acids of all types, proteins, lipids, cytokines, and other soluble factors. Exosomes may be taken up by cells in their vicinity or in a distant site. Since the cargo of exosomes is composed of biologically active molecules, they serve as mediators of cell–cell communication. As such they may impose phenotypic changes, including extracellular processes and even morphological changes in the recipient cells. In this way secreted exosomes communicate with tumor cells to promote carcinogenesis, tumor growth and survival, drug resistance development, and metastasis formation [7,8,9].

The uptake of CLL-derived exosomes by adjacent cells may promote disease progression by affecting the function of stroma cells, including division, migration, and secretion of cytokines associated with inflammatory processes [1,10].

Studies have shown that at least 50% of exosomes in the serum of patients with CLL originate from CLL cells [7,11]. A recent study showed that exosomes derived from bone marrow (BM) mesenchymal stromal cells caused a decrease of spontaneous apoptosis of leukemic cells and an increase in their chemoresistance to several drugs, including fludarabine ibrutinib, idelalisib, and venetoclax [12], demonstrating that exosomes play a crucial role in CLL B cells/BM microenvironment communication. Another recent study showed that plasma-derived exosomes from patients with CLL exhibit different protein cargo compositions depending on disease status and progression. Along these lines, we have shown that CLL cells secrete exosomes which, after engulfing in endothelial cells, initiate a viscous circle that involves the secretion of IL-6 by these cells which, when it is taken up by the neoplastic cells, protect from apoptosis [13].

Monocytes and their progeny form a significant part of lymphoproliferative disorders, including CLL. Resistance to apoptosis in CLL is mainly attributed to the ability of the malignant cells to shape their microenvironment for an immune and tumor-supporting niche, and myeloid cells are considered key players in this regard [14]. CLL cells modulate monocytes to become nurse-like cells (NLC) and fibroblast-like cells called fibrocytes [15]. These cells promote lymphocyte proliferation and survival, confer resistance to chemotherapy, and are associated with a more rapid disease progression, e.g., by producing connective tissue proteins such as vimentin and collagens [14,16,17]. The interplay between CLL cells and monocytes is also demonstrated by the high number of fibrocytes present in the BM of CLL patients. CLL cells trigger the release of CCL2 or CD14 from monocytes, thus stimulating an inflammatory environment which supports the tumor [18]. In spite of the above-mentioned studies, the mechanism by which CLL cells induce the differentiation of some monocytes into fibrocytes is still unknown. To close this knowledge gap, we aimed to understand whether and how CLL-derived exosomes modulate the phosphoproteome landscape of monocytes, thus affecting their phenotype to become CLL-supportive cells.

## 2. Results

### 2.1. Isolation of Monocyte and B-CLL Cell

**Figure 1:**
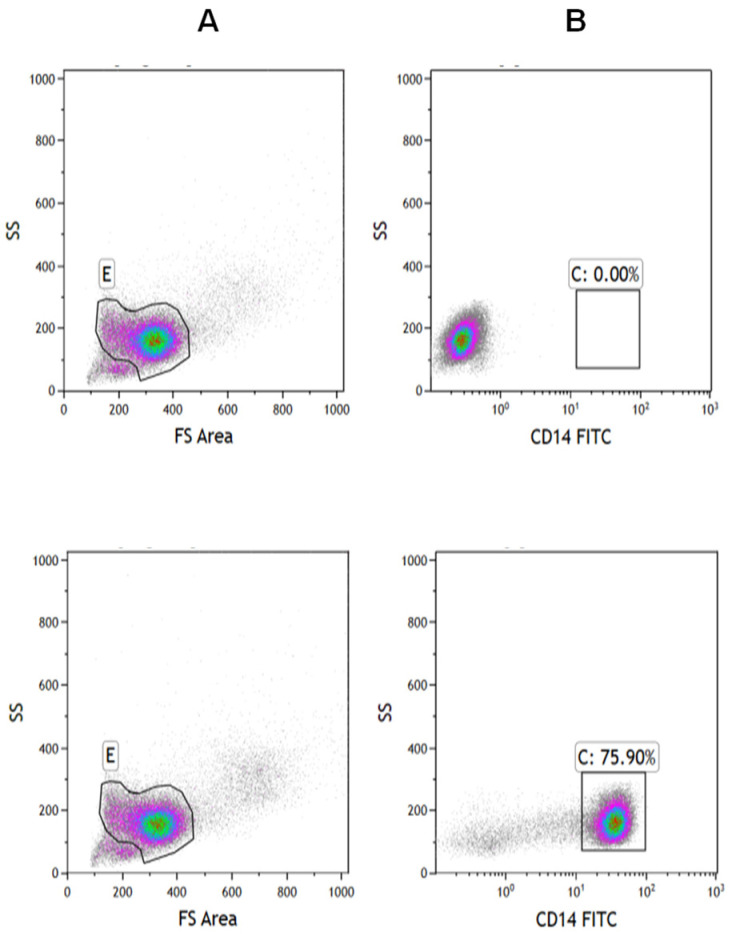
Validation of the quality of isolation of monocytes by flow cytometry. Cells were isolated using the RosetteSep™ isolation kit and stained for the anti-human CD14-FITC antibody: (**A**): Density plot of side and forward scatters of control and stained cells; (**B**): Density plot of side scatter of control and CD14-FITC: (0%, 75.90%) stained cells out of E gate; The experiment was repeated three time with similar results.

**Figure 2:**
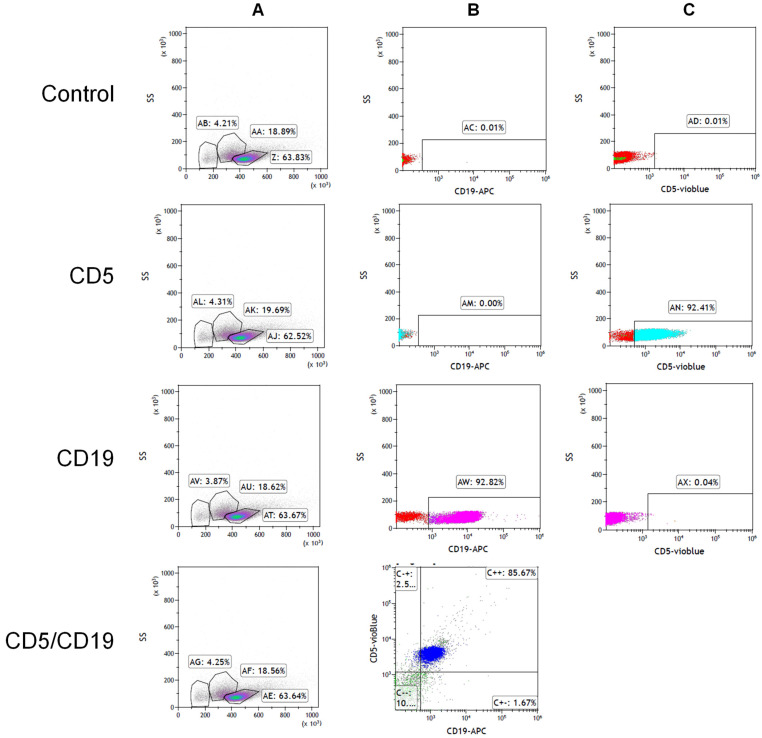
Validation of the quality isolation of B-CLL cells by flow cytometry. Cells were isolated using the Ficoll-Paque™ density gradient and stained for CD19-APC and CD5-Vioblue antibodies: (**A**): Density plot of side and forward scatters of control and stained cells, Z, AJ, and AT; (**B**): Density plot of side scatter of control and stained cells CD19-APC (0.01%, 0%, and 92.82%); (**C**): Density plot of side scatter of control and stained cells CD5-Vioblue (0.01%, 92.41%, 0.04%). This analysis was repeated three times with reproducible results.

We isolated monocytes from the blood samples of healthy volunteers and the B-CLL cells from the patients’ samples. To verify the validity of the isolation, we analyzed the cells by flow cytometry using the relevant antibodies. Figure 1 shows the isolated monocytes (CD14 positives) which constituted 75.90% of the cells. Figure 2 shows the isolation results of the B-CLL cells (CD5 and CD19 positives). The B-CLL cells constituted 92% of all cells.

### 2.2. Isolation of Exosomes

**Figure 3:**
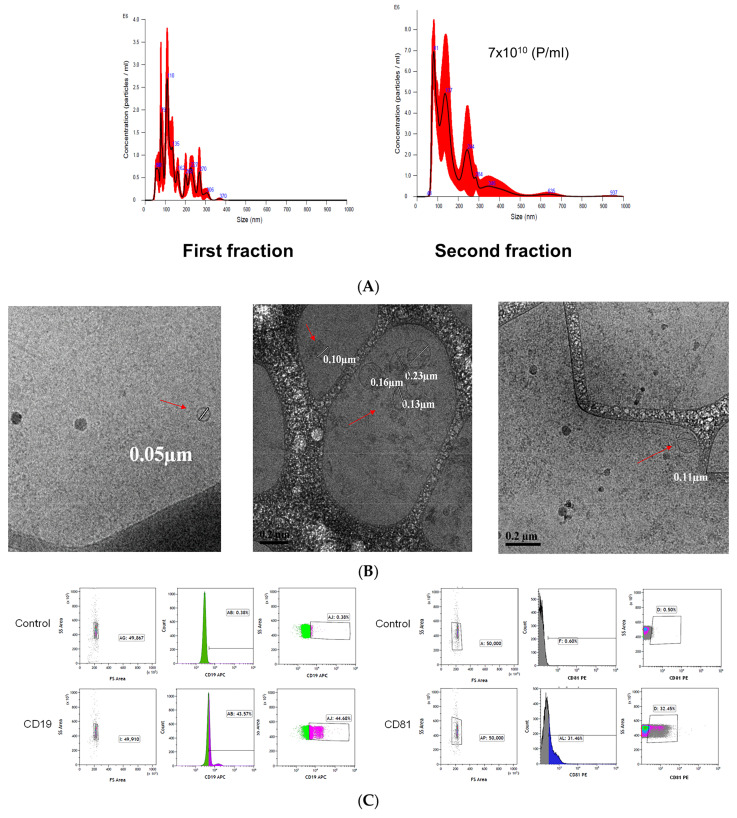
Validation of the quality isolation of exosomes by NTA, TEM, and FACS. Exosomes were isolated by centrifuging and stained for the CD19-APC antibody. (**A**): NTA output of our exosomes. (**B**) TEM analysis of the isolated exosomes, arrowheads point to the vesicles. (**C**) Upper panel: Density plot of side and forward scatters of control and stained particles; (**Left panel**): Histogram of side scatter of CD19-APC-stained particles (0.39%, 43.58%); (**Right panel**): Density plot of side scatter of particles stained with CD81-APC (0.38%, 44.68%). These analyses were repeated four times obtaining similar results.

To isolate the CLL-derived exosomes, we harvested PBMCs from 45 treatment-naïve patients (see Table 1 for the patients’ characteristics). The CLL-derived exosomes were extracted from culture media of 20–40 × 10^6^ CLL cells/mL that was collected 72 h after cell seeding by differential ultracentrifugation. To verify that the isolation was successful, we used three analyses: Nano-Sight Tracking Analysis NTA, TEM, and flow cytometry with anti-human CD19, CD81, and CD5 antibodies (Figure 3A).

NTA quantification of the two fractions of purified exosomes, both of Pellet’s pellet (“pellet”) and from supernatant’s (“sup”) pellet revealed that the microparticles were of a high concentration and at the expected size of the exosomes, which ranged from 30 to 150 nm in diameter. The second (“sup”) fraction was used for further experiments, since it contained more exosomes than the first (“pellet”) one. For further characterization of the exosomes, we used transmission electron microscopy (TEM) imaging analysis (Figure 3B). To show that the isolated particles are exosomes, we stained them with anti-human CD81 cells, and to show that these exosomes are of B cells of origin, we stained them with anti-human CD19 cells.

### 2.3. Uptake of Exosomes

**Figure 4:**
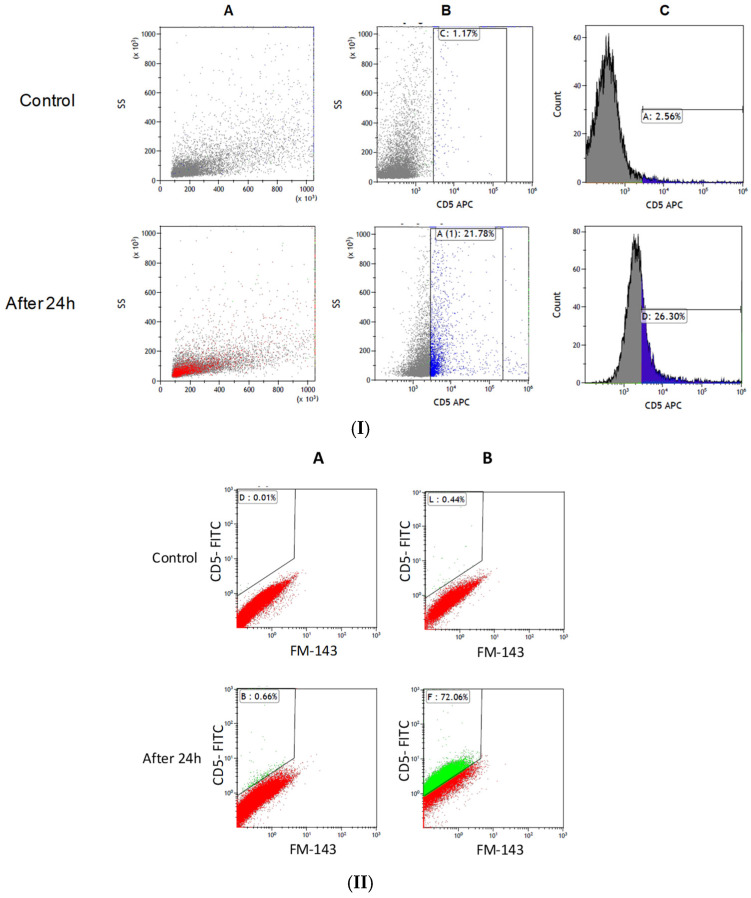
Uptake of CLL-derived exosomes by monocyte cells 24 h after exosomal exposure. Monocytes were exposed to CLL-derived exosomes and the exosomal uptake was measured by flow cytometry. (**I**) Single exosomal labeling: A: Density plot of side and forward scatters of control and exposed cells; B: Density plot of side scatter of control and exposed cells stained with CD5-APC (1.17%, 21.78%); C: Histogram of side scatter of control and stained cells CD5-APC (2.56%, 26.30%). Control cells: cells that were not exposed to exosomes. (**II**) Double exosomal labeling using FM-1-43 exosomal dye and CD5 coupled to FITC. FM-1-34-stained exosomes were applied to the isolated monocyte cells that were subsequently stained with anti-human CD5-FITC antibodies. As before, four repeated analyses showed similar results for the uptake experiments as well. A,B—a similar analysis as above, using double stained vesicles.

Monocyte cells were exposed to 1.4 × 10^10^ CLL-derived exosomes for 24 h. Then, cells were collected and the exosomal uptake was measured by flow cytometry. Figure 4 shows the percentage of exosomal uptake by the monocyte cells. After 24 h, 21.78% of the population contained the typical CLL-derived exosome marker (CD5), demonstrating a CLL-derived exosomal uptake. Although the extent of the uptake was relatively mild, it induced a profound effect on the recipient monocytes, as shown below.

### 2.4. Monocytes Acquire Fibrocytes Morphology in Response to CLL-Derived Exosomes

Some monocytes acquire an in vivo fibrocyte-like morphology during the disease of CLL [19]. These fibrocyte-like cells are considered “nurse like cells” that secrete a variety of cytokines to support the growth of CLL cells [19]. To study whether exosomes secreted from the neoplastic cells promote this differentiation, we exposed monocytes to CLL-derived exosomes.

**Figure 5:**
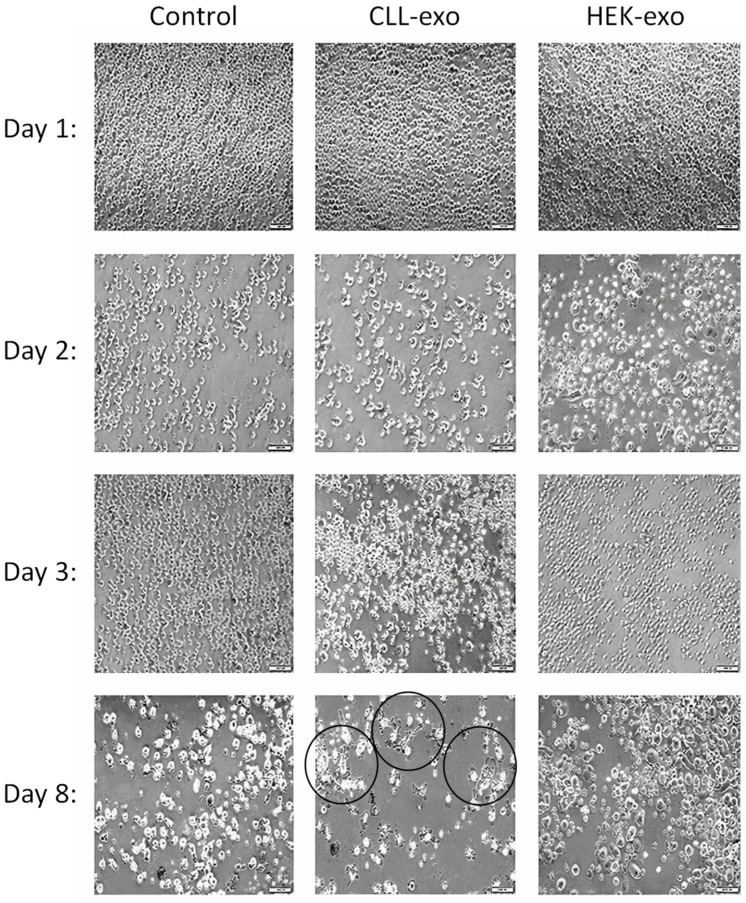

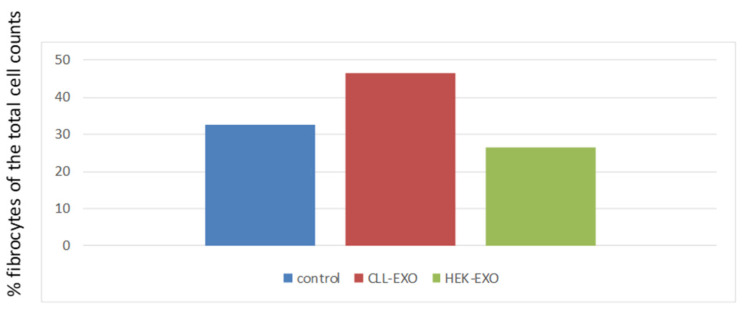
Percentage of morphologically altered monocytes after 8 days of exosomal exposure. (**Upper panels**): microscopic images of cells at the indicated days, fibrocytes are circled; (**Lower panel**): quantification of the amounts of fibrocyte cells in %. The experiment was repeated once more and demonstrated similar results. Fibrocytes are circled.

Monocytes were isolated from whole blood (Magen David Adom) and were exposed to CLL-derived exosomes for 24 h. As a control, these monocytes were exposed also for a similar number of exosomes isolated from HEK cells. Another group of monocytes were left intact (no exosomal exposure) as another control. The morphology of the cells was followed by inspecting the cells using an inverted microscope every few days. The results are shown in Figure 5. Four days after exposure, we detected a change in the cells’ morphology. The cells became less round and more “elongated” like fibroblasts (circled in three spots). Exposure to the CLL-derived exosomes resulted in the highest number of transformed cells.

**Figure 6:**
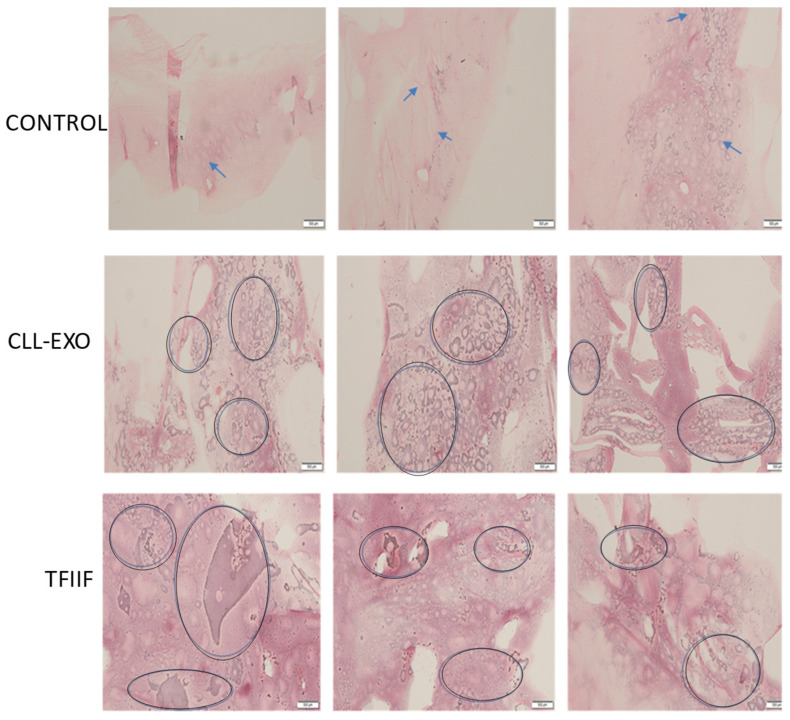
Monocytes are transformed into reticulin-producing cells after exosomal exposure or ectopic expression of TFIIF. Microscopic images of reticulin-stained monocytes (fibrocytes), marked by circles, treated with CLL-derived exosomes 6 days after exosomal exposure or ectopic expression of the TFIIF gene. Arrowheads depict several reticulin-secreted cells among the control unexposed cells. The secretion of reticulin typifies fibrocytes that were transformed from normal monocytes in response either to CLL exosomes or to the ectopic expression of the TFIIF gene. A size ruler is indicated at the bottom right of each. This scale bar is 100 nm.

To prove that the transformed cells are fibrocytes, we looked for cells that produce reticulin, which typifies fibrocytes. As shown in Figure 6, the cells that have become fibrocytes, mesenchymal cells, secreted reticulin, depicted by their specific positive staining with reticulin.

### 2.5. Phosphoproteomic Profiling

To study to what extent monocytes were affected by the CLL-derived exosomes in terms of protein phosphorylation, we used protein extracts of the monocytes with or without the CLL-derived exosomes that were collected for mass spectrometry analysis followed by the phosphoproteomic profiling. Table 2 shows the resulting change in the phosphorylation state in 67 proteins. The proteins are arranged in an “S” according to the extent of the change, the first being the protein with the largest change in its phosphorylation status between the treated and control cells. We choose to focus on two proteins that were the top phosphorylated proteins in our setting and were biologically relevant to our study topic, T2FA (-TFIIF) (#5) and NOG1 (-GTPBP4) (#3).

**Figure 7:**
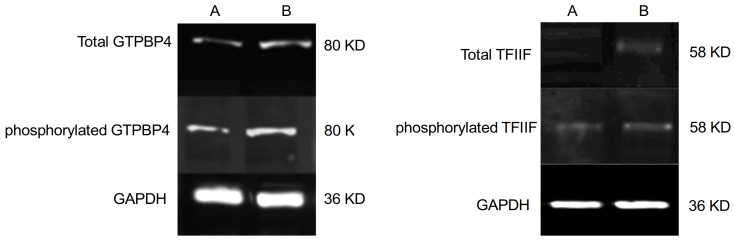
Validation of the phosphoproteomic results. (**Left panel**): total GTPBP4 and phosphorylated GTPBP4. Column A: Control (non-exposed monocyte cells); Column B: Monocytes exposed to exosomes for 24 h. (**Right panel**): total TFIIF and phosphorylated TFIIF. Column A: Control (non-exposed monocyte cells); Column B: Monocytes exposed to exosomes for 24 h.

Validation of the phosphoproteomic results was performed by following the phosphorylation status of the above-mentioned two proteins by Western blotting. Monocyte cells were exposed to CLL-derived exosomes for 24 h, followed by protein extraction. Proteins were then immunoprecipitated by their specific antibodies, followed by Western blotting with anti-phosphoserine and secondary fluorescent antibodies. Indeed, an increase in the phosphorylation status of the two proteins was obtained. GTPBP4 increased 1.72 times compared to control; similarly, its phosphorylated form increased 1.81 times (Figure 7 lower panel). The TFIIF protein increased 3.7 times compared to control, while the phosphorylated protein increased 4.53 times (Figure 7 upper panel).

### 2.6. The Effects of CLL-Derived Exosomes on IL-6 Secretion and STAT3 Phosphorylation in the Neoplastic CLL Cells

**Figure 8:**
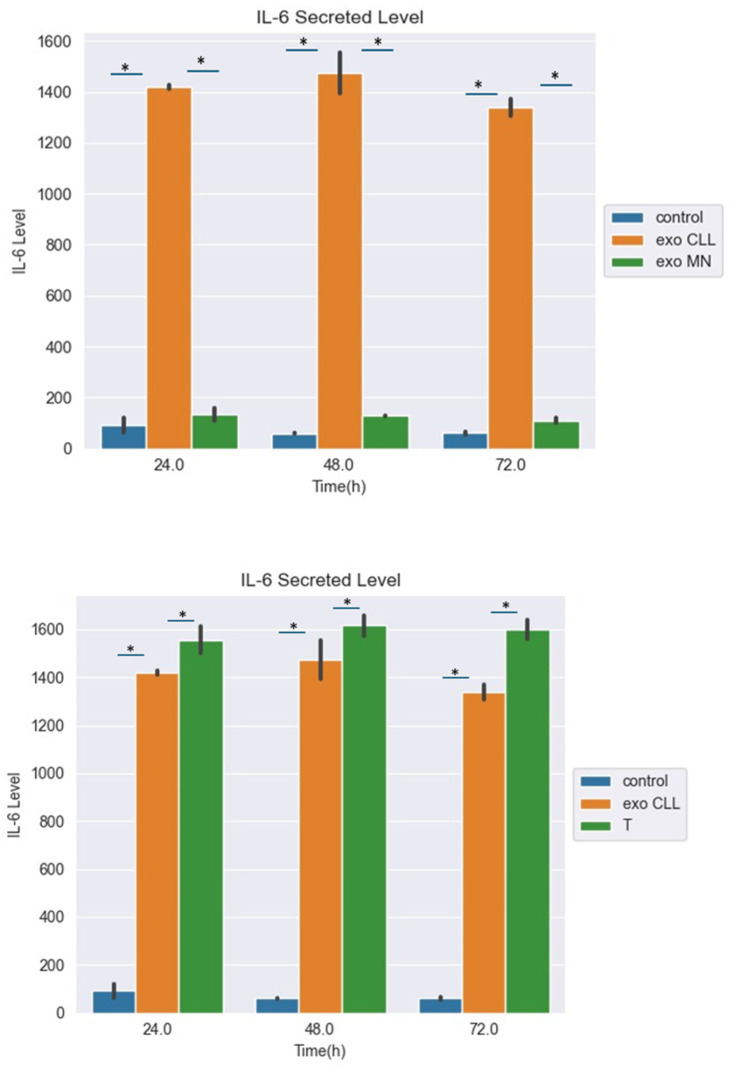
ELISA analysis of IL-6 secreted levels (pg/mL) by monocyte cells in various conditions. (**Upper panel**): monocyte cells were exposed either to CLL-derived exosomes (exo CLL), healthy mononuclear exosomes (exo MN), or to PBS alone (control). The growth media was collected and subjected to IL-6 level measurements during a period of 24 h, 48 h, and 72 h by using the Human IL-6 Quantikine ELISA Kit. (**Lower panel**): similar ELISA-based analysis of monocytes that were transfected with a TFIIF open reading frame containing plasmid. IL-6 levels were tested during a period of 24 h, 48 h, and 72 h, Monocytes exposed to the transfection buffer (Control); monocytes transfected with the TFIIF (T) gene, monocytes with CLL exosomes (exo CLL). Asterisks depict statistical significance (*p* < 0.05).

Monocytes support the growth of CLL cells by secreting cytokines [20]. However, how CLL cells signal monocytes to secrete these cytokines is not yet known. We hypothesized that this is achieved by exosomes secreted from the neoplastic cells. Therefore, we next studied the effects of the CLL-derived exosomes on possible secretion of IL-6 by exposed monocytes. In this experiment, IL-6 levels were tested during a period of 24 h, 48 h and 72 h, by using the Human IL-6 Quantikine ELISA Kit (Figure 8). We used monocyte cells divided into three groups: normal monocytes (control), monocytes with CLL-derived exosomes (exo CLL), and monocytes with healthy mononuclear exosomes (exo MN), for another control group, to assess the specificity of the activity of the CLL-derived exosomes.

Cells in all groups were grown under the same conditions. Figure 8 shows that the IL-6 levels in the growth medium were significantly higher in monocytes treated with CLL-derived exosomes compared with the two other controls.

After inspecting the increase in IL-6 secretion by monocytes in response to the CLL-derived exosomes, we wanted to test whether the chosen protein, TFIIF, also induced IL-6. For this purpose, monocyte cells were transfected with the TFIIF gene [Figure 8, right graph (T group)]. The IL-6 levels in the growth medium were significantly higher in the monocytes treated either with the CLL-derived exosomes or transfected with the TFIIF gene compared to the control group. The TFIIF-transfected cells secreted the highest levels of IL-6 in our setting.

### 2.7. CLL Cells Induce the Phosphorylation of STAT3 in Response to IL-6 Secreted by Pre Stimulated Monocytes with CLL-Derived Exosomes

Previous studies have shown that IL-6 is able to induce the activation of STAT3 by increasing its phosphorylation status in CLL cells [6,21].

**Figure 9:**
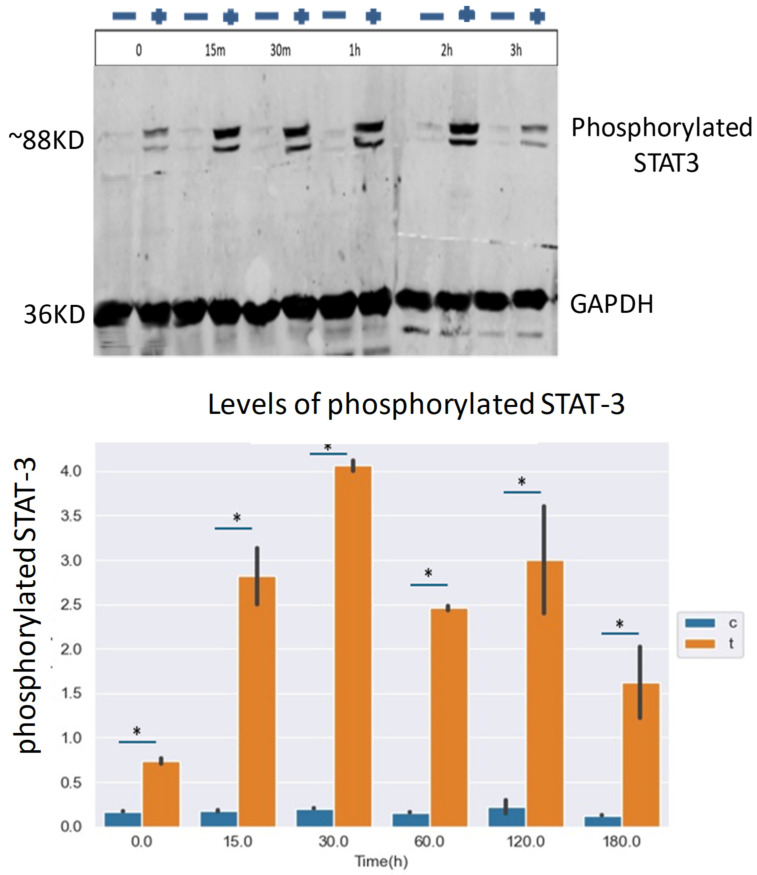
STAT3 is phosphorylated in CLL cells in response to IL-6 produced by CLL-derived exosomal exposed monocytes. Intact CLL cells were exposed to the conditional medium of monocytes pre-exposed to CLL-derived exosomes, and STAT3 was assessed by Western blotting. + and − represent with or without exosomes. (**Upper panel**): an example of a representative blot; (**Lower panel**): quantification of three independent experiments. Control cells exposed to PBS (C), CLL cells exposed to monocyte conditional medium (T). * depicts statistical significance (*p* < 0.05).

In this experiment, we isolated monocytes and exposed half of them to CLL-derived exosomes. After 72 h, the growth medium was collected and added to the neoplastic intact CLL cells. The phosphorylation of STAT3 was assessed 0–180 min after the conditional medium exposure. STAT3 was subsequently immunoprecipitated, and its phosphorylation status was assessed by Western Immunoblot. Figure 9 shows the Western immunoblot results, depicting a marked increase in the phosphorylation status of STAT3 in response to the conditioned medium of the monocytes exposed to the CLL-derived exosomes. The pick of phosphorylation was obtained 15 min after medium exposure. This phosphorylation demonstrates the vicious circle induced by CLL-derived exosomal secretion.

**Figure 10:**
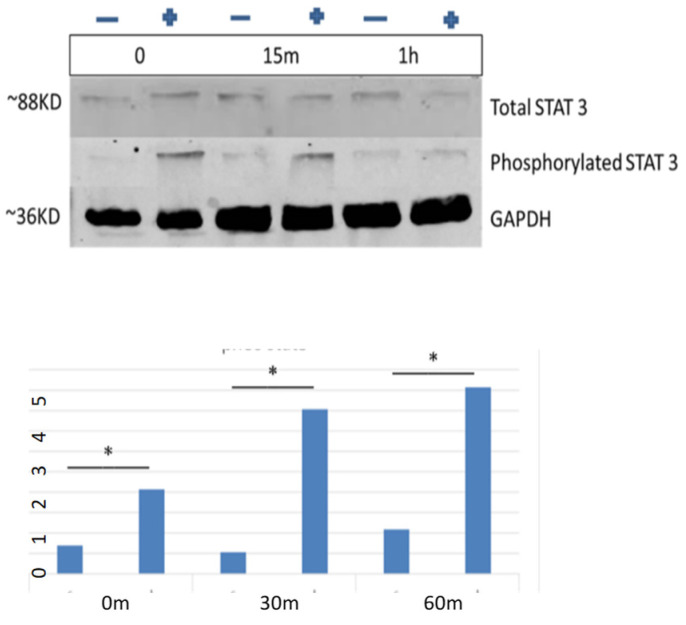
STAT3 is phosphorylated in CLL cells in response to IL-6 produced by TFIIF transfected monocytes. Intact CLL cells were exposed to the conditional medium of monocytes that were transfected with the TFIIF gene, and the levels of STAT3 were assessed by Western blotting. + and – represent with or without exosomes. (**Upper panel**): an example of a representative blot; (**Lower panel**): quantification of three independent experiments. Note: * denotes statistical significance (*p* < 0.01).

Subsequently, we wanted to test whether TFIIF is able to induce the phosphorylation of STAT3 as well. The same experiment was repeated, but this time the treatment group included monocytes that were transfected with the TFIIF gene. Figure 10 shows the Western immunoblot results. Phosphorylated STAT3 increased 13.2-fold at time 5 min compared to control, 9.86-fold 15 min after exposure, and 5.6-fold 1 h after exposure to the conditional medium.

## 3. Discussion

CLL cells interact with various cell types, such as monocytes, mainly by “reprogramming” cellular processes [3,5,12]. Various recent studies have shown the dependency of neoplastic cells in their microenvironment inducing disease progression [22,23]. Our results shed some more light on this phenomenon. Here, we show that CLL-derived exosomes (and not HEK293-derived exosomes) specifically induced morphological and functional changes in a portion of the exposed monocytes to become NCL and fibrocytes. Such fibrocytes are known to be able to secrete many cytokines that support the growth of CLL [15,16]. These modulated cells were able to secrete reticulin, a connective tissue marker, confirming their transformation into fibrocytes. Of note, the results of our study were obtained in spite of the potential heterogeneity both of the CLL patients and of the healthy donors. Interestingly, Mesaros et al. showed that this mode of monocytes, which are differentiated into macrophages, are referred to as tumor-associated macrophages (TAMs). TAMs secrete cytokines and chemokines, exerting an anti-apoptotic, proliferative, and pro-metastatic effect on tumor cells [20].

Along these lines, studies have shown that macrophages, dendritic cells, neutrophils, myeloid-derived suppressor cells, mast cells, eosinophils, and basophils act alone or in concert to shape tumor cell resistance through cellular interaction and/or release of the soluble factors favoring survival, proliferation, and migration of tumor cells, including immune-escape and therapy resistance (reviewed in ref. [24]).

A previous study conducted in our laboratory showed that exosomes derived from leukemic T cells secreted the transcript of human telomerase (hTERT). These exosomes were subsequently taken up by fibroblast cells in which hTERT was translated into a fully active enzyme, thus promoting numerous phenotypic changes, including increased cell proliferation, delayed cellular senescence protection against apoptosis, and partial protection from DNA damage [25].

The transformation of monocytes to fibrocytes via exosome shuttling demonstrates how CLL cells recruit monocytes to acquire survival advantage. We have further showed here that CLL-derived exosomes induced monocytes to secrete IL-6. Along these lines, we have shown in a parallel experiment conducted in our laboratory that these exact exosomes secreted by CLL cells were also able to induce endothelial cells to secrete IL-6 as well as other cytokines, demonstrating a global ability of these exosomes to gain survival and growth support by recruiting bystander cells for their benefit [21].

A phosphoproteome analysis of monocyte cells exposed to CLL-derived exosomes revealed that CLL-derived exosomal exposure increased the phosphorylation of 67 proteins, underlying the activation of signal transduction pathways that may affect cellular phenotypes following various stimuli. Such changes were demonstrated by us in a previous study in our laboratory, which described changes in protein phosphorylation following exposure of endothelial cells to exosomes secreted from CLL cells [21]. Along these lines, exosomes secreted by cervical cancer cells have activated ERK and AKT pathways in recipient epithelial cells by increasing the phosphorylation of proteins in this pathway [26]. In another example, metabolic changes occurring in cells following exosome uptake were shown to be mediated by changes in the phosphorylation of specific proteins [27]. In an attempt to identify the key protein that may mediate the effects of CLL-derived exosomes on monocytes, we have chosen TFIIF, one of the top proteins in which its phosphorylation form was upregulated in our monocytes in response to CLL-derived exosomes. TFIIF is a general transcription factor which is a part of the RNA polymerase II pre-initiation complex [28]. Therefore, we expressed ectopically the TFIIF open reading frame in monocytes and assessed the resulting features thereafter.

Hence, upon TFIIF ectopic expression in our monocytes, we obtain a similar induction of the secretion of IL-6 into the growth medium, suggesting that phosphorylated TFIIF may mediate the CLL-derived exosomal-dependent IL-6 secretion. The difference in the maximal secretion time of IL-6 in response to CLL-derived exosomes versus the ectopic expression of TFIIF (24 and 48 h, respectively) suggest that the IL-6 secretion might be mediated by other processes as well, and not solely by TFIIF. Previous studies have found that IL-6 increases the level of STAT3 activity by inducing its phosphorylation [6,21]. In addition, human recombinant IL-6 was shown to induce phosphorylation of STAT3 in a dose-dependent manner in CLL cells, which in turn activates pro-survival pathways [6].

Our study suffers from various limitations. Firstly, there is a lack of an in vivo validation, an issue that is addressed in a subsequent study in our laboratory. Fibrocytes were characterized only by inspecting morphological changes and the secretion of reticulin, not by flow cytometry. The validation of our suggested mechanism may gain from additional interventions, such as the inhibition of Il-6 production or downregulation of the TFIIF gene product. Additionally, the suggested survival advantage resulting from STAT3 activation in the neoplastic cells was not presented here; however, in a parallel study conducted in our laboratory [13], we presented evidence of decreased apoptosis due to STAT3 activation in the same CLL cells. Another point to consider is that, instead of the HEK293-derived exosomes that were used as a negative control, maybe we should have used PBMCs instead. As stated above, these drawbacks will be addressed in future studies. Finally, monocyte purity was only ~76%, which may raise a question about the source of the secreted cytokines. However, the fact that a portion of these monocytes was transformed into fibrocytes that are known to secrete cytokines supports our conclusions of the vicious circle initiated by CLL-derived exosomes that ultimately provide them with a survival advantage.

All in all, our study aimed at helping to close the knowledge gap regarding the contribution of monocytes to the aggressiveness of CLL as mediated by exosomes derived from neoplastic cells. A vicious circle is initiated by CLL cells through the secretion of exosomes that are taken up by monocytes that secrete cytokines which subsequently induce the upregulation of STAT3 in the neoplastic cells, thus providing them a survival advantage.

## 4. Materials and Methods

### 4.1. Patients’ Characteristics

Peripheral blood samples were obtained from 45 treatment-naïve patients diagnosed with CLL who were followed at Rabin Medical Center after signing informed consent forms. This study received the approval of our Institutional Review Board (072-17-RMC).

### 4.2. Source of B-CLL

This study was approved by the Beilinson Review Board. Blood was donated by CLL patients who signed informed consent forms before recruitment to the study. Each blood sample contained white blood cells between 10 and 200 × 10^3^ cells/μL, while at least 70% of each blood count were considered to be CLL cells. These cells were used throughout the study according to each specific experiment. Experimental repeats were usually three, as specified in each figure legend. Samples were analyzed individually.

### 4.3. Isolation of B-CLL

From each blood sample, CLL cells were isolated (maximum 24 h after blood donation) by using Ficoll-Paque™ density gradient centrifugation, according to the manufacturer’s protocol. Then, 7–20 × 10^6^ cells/mL were cultured for 72 h in 37 °C, 5% CO_2_, and 90% humidity, in RPMI-1640 medium (biological industries, Beit HaEmek, Israel), containing 10% of exosome-free fetal bovine serum (FBS), 1% L-glutamine, and 1% penicillin-streptomycin (Biological Industries, Beit HaEmek, Israel). Exosomes were removed from the FBS following an ultracentrifugation for 18 h at 100,000 RCF at 4 °C.

### 4.4. Isolation of Monocyte Cells

Monocyte cells were isolated from Buffy coat samples of mononuclear leukocyte cells obtained from the Israeli Blood Bank (Magen David Adom, Shaba Medical Center) by the RosetteSep™ Human Monocyte Enrichment Cocktail (ENCO Scientific Services, Petah Tikva, Israel), according to the provided protocol followed by an additional Ficoll density gradient procedure.

### 4.5. Exosome Isolation

An amount of ~20–40 × 10^6^ CLL cells/mL cells were cultured, and 72 h later their growth medium was centrifuged at 1000× *g* for 10 min. Then the supernatant was centrifuged at 2000× *g* for 10 min, followed by 10,000× *g* centrifugation for 30 min at 4 °C. Afterwards, the supernatant was filtered using 0.22 μm filters, and was then ultracentrifuged at 100,000× *g* for 70 min at 4 °C. Subsequently, the supernatant was removed and the pellet was washed with PBS (Dulbecco’s Phosphate-Buffered Saline, without Calcium and Magnesium, Biological Industries, Beit HaEmek, Israel) by ultracentrifuge for 120 min at 100,000× *g*, 4 °C. The supernatant phase was centrifuged as well. Then, the supernatant was removed, and the pellet was washed and resuspended with 200 μL PBS and kept at −80 °C until further use.

### 4.6. Assessment of the Purity and Concentrations of the Isolated Exosomes

The purity of exosomes by size (30–150 nm) and concentrations (particles/mL) were performed by using the Nano-Sight tracking Analysis at the Weizmann Institute of Science (Rehovot, Israel). The transmission electron microscope (TEM) imaging was inspected to see and define the shape of the isolated vesicles. To explore the source and identity of our exosomes, flow cytometry using fluorescent antibodies against exosomal (CD81) and hematological (CD5, CD19) markers was used.

### 4.7. Flow Cytometry Analysis

Samples were immunostained with the relevant anti-human CD protein antibodies expressed on the membranes of each cell type: α CD5-APC, CD19-APC antibodies for B-CLL, α CD14-FITC antibodies for monocyte cell, and α CD81 typifying exosomes according to the provided protocol (Miltenyi Biotec, Bergisch Gladbach, Germany). For double staining of the exosomes, we labeled them firstly with FM-1-43 exosomal dye (Thermo Fisher Scientific, Waltham, MA, USA), according to the provided manual, and subsequently cells were stained with α CD5-FITC prior to the flow cytometry analyses. To exclude non-specific binding of the antibodies, samples were also labeled with an isotype control. Flow cytometry was performed using the Gallios^®^ Flow Cytometer (Beckman Coulter International SA, Switzerland). Data analysis was performed using the Kaluza Acquisition Software 2.1 (Beckman Coulter International SA, Switzerland).

### 4.8. Cell Culture

A total of 5 × 10^6^ of human monocytes were plated in 6 well plate with 3 mL of high glucose RPMI-1640 medium (Biological Industries, Beit HaEmek, Israel), containing 10% exosome-free fetal bovine serum in 37 °C 5% CO_2_ for 2 h, then replaced with a new medium. Cells were treated as follows:

(1) After 24 h, B-CLL exosomes were added to the plate for 24, 48, and 72 h in 37 °C at 5% CO_2_. The medium was collected at various time periods (24, 48, and 72 h), and saved for further experiments.
(2) After 24 h, B-CLL exosomes were added to the plate for 7 days. On each day, the cells were photographed under the inverted microscope to track possible morphological change in response to CLL-derived exosomal exposure.

### 4.9. Mass Spectrometry and Phosphoproteomic Analysis

Protein samples (1 mg/mL per repeat) of cell cultures (monocyte with exosome) were collected for mass spectrometry analysis followed by the phosphoproteome profile analysis, all performed at the Proteomics Unit, The Weizmann Institute of Science.

### 4.10. Immunoprecipitation (IP)

Validation of selected phosphoproteins was performed by IP and Western immunoblotting. A total of 40 μL of protein AG Sepharose beads were washed twice with 500 μL lysis buffers (pH = 8) at 2000 RPM for 1 min. The beads were immersed in 1 mL lysis buffer and the antibodies against the chosen proteins, GTPBP4 and TFIIF (Abcam, Cambridge, UK), used according to the recommended concentrations, were incubated for 4 h at 40 °C with agitation. Then, 500 μL lysis buffer was added and the beads were washed twice at 2000 RPM for 1 min. A 1000 μL lysis buffer containing proteasome and phosphatase inhibitors [0.5 mg/mL protease inhibitor (PI), 100 mM dithiothreitol (DTT), 40 mM phenylmethylsulfonyl fluoride (PMSF), 1 M sodium fluoride (NAF), and 50 mM sodium orthovanadate (SOV)] were added to 1 mg of monocyte protein extracts (protein concentration was measured by the Bradford reagent assay), after CLL-derived exosomal exposure for 24 h and incubated for 16 h at 40 °C with agitation. Afterwards, the samples were spanned-down, centrifuged at 2000 RPM for 1 min and washed three times with 500 μL lysis buffer at 2000 RPM for 1 min. Then, each pellet was immersed with 24 μL lysis buffer with the addition of 6 μL sample buffer, boiled for 3 min, and loaded on 10% sodium dodecyl sulfate polyacrylamide gel electrophoresis (SDS-PAGE) gels for Western blot analysis.

### 4.11. Western Immunoblotting

A total of 1 mg of protein extract was subjected to SDS-PAGE and transferred to a nitrocellulose membrane. The membrane was then hybridized for 16 h at 40 °C (with agitation) with anti-phosphoserine antibodies (Abcam) to detect the phosphorylated form of the GTPBP4 and TFIIF proteins, using the recommended antibody concentrations in the manufacturer’s manuals. On the following day, the membranes were subjected to fluorescent labeled secondary antibodies. Visualization was performed by the Odyssey analysis software 6.0 (Odyssey IR imaging system; LI-COR, Lincoln, NE, USA). Total protein levels of GTPBP4 and TFIIF were evaluated by Western immunoblotting, as described above. Visualization and expression level measurements were performed by using the Odyssey analysis software (Odyssey IR imaging system; LI-COR).

### 4.12. Bacterial Growth

*E. coli* gram negative bacteria harboring a plasmid containing the open reading frame of human GTF2F1 (TFIIF) (Sino Biological, BDA, Beijing. China.), were grown on a Petri dish containing Luria Broth (LB) agar with kanamycin (50 µg/mL) for 24 h at 370 °C. Then, the bacteria were transferred to a liquid agar (1:4) in Erlenmeyer flasks and cultured for another 24 h at 37 °C, with agitation, prior to plasmid isolation.

### 4.13. Plasmid Isolation

Plasmid isolation after the bacterial growth was performed using the NucleoBond^®^ Xtra midi plus kit (Macherey-Nagel Inc., Allentown, PA, USA), according to the provided protocol.

### 4.14. DNA Concentration

DNA concentration of the plasmids was measured by the Nano-drop(Thermo Scientific, Waltham, MA, USA) apparatus (ratio of DNA wavelength absorption of 260/280 was between 1.8 and 2.0).

### 4.15. Bacterial Transfection

Transfection of monocytes with the TF2F gene containing plasmid was performed using the Lonza Amaxa™ Human Monocyte Nucleofector Kit, according to the provided protocol.

### 4.16. Validation of the Transfection Efficiency

Validation of monocyte transfection with TF2F gene was performed by assessing the protein level by Western immunoblot (using anti-TF2F, Abcam) 24 h after transfection. In addition, we also examined the green fluorescent protein (GFP) containing plasmid transfection under the fluorescence microscope as a control for the transfection efficiency.

### 4.17. ELISA Assay

The secretion of Interleukin 6 (IL-6) to the media was measured by the Human IL-6 Quantikine ELISA Kit (R&D Systems, Minneapolis, MN, USA), according to the provided protocol.

### 4.18. Reticular Fiber Detection

Cells were stained with Gomori’s silver plating (a kit of reticulum stains (modified Gomori)), (Bio Optica Milano Spa, Milan, Italy) according to the standard research protocol. After staining, cells were inspected under the light microscope.

### 4.19. Statistical Analysis

Data are presented as average ± standard error mean (SEM). Student’s *t*-test was used to calculate statistical significance. The analysis was performed using the GraphPad Prism 8 version.

## 5. Conclusions

In summary, our study shows that monocyte cells take up exosomes that subsequently increased the phosphorylation status of 67 proteins. This increase induces phenotypic changes in the recipient monocytes, transforming them into fibrocytes that support the survival of neoplastic cells by secreting IL-6. The secretion of IL-6 phosphorylates STAT3 in the neoplastic CLL cells, thus potentially providing them with a survival advantage. The effects of CLL-derived exosomes on bystander monocytes may be mediated, at least in part, by TFIIF. The vicious circle, described in our study, initiated by CLL cells, presents an important layer of communication between CLL cells and their bystander monocytes to acquire a survival advantage in an exosome-dependent way. Our study needs further validation to clarify the microenvironmental mutual effect contributing to the aggressiveness of CLL, and studies currently conducted in our lab are directed to resolve these issues.

## Figures and Tables

**Table 1:** Baseline patient characteristics (*n* = 45).

Characteristics	Measure/Category		Overall
Age/years	Median (range)		71 (52–89)
White blood cells ×10^9^	Median (range)		66 (14–292)
Absolute lymphocyte count	Median (range)		54 (9–281)
**Rai stage**	N (%)		
0			27 (60)
1			7 (16)
2			6 (13)
3			1 (2)
4			4 (9)
β2M (mg/L)	Median (range)		2.807 (1.646–8.020)
β2M (mg/L)	<4/≥4/unknown		23/10/12
**IGHV mutation**	N (%)		
Mutated			10 (22.2)
Unmutated			20 (44.4)
Unknown			15 (33.3)
**FISH results, N**	Del 17p/11q/tri12/13q/negative/unknown		3/4/5/15/4/19
**Characteristics**		**Data**	
**Comorbidities N (%)**			
Ischemic heart disease		10 (22)	
Diabetes Mellitus		9 (20)	
Hypertension		4 (9)	
Cerebrovascular accident		0 (0)	
**Patients with other malignancies N (%)**		9 (20)	
**Type of other malignancies (N)**		6	
**Mean ejection fraction (%) (S.Dev.)**		58 (9.7)	
**Drugs N (%)**		24 (53)	
Aspirin		18 (40)	
Statins		20 (44)	
Metformin		7 (15)	

Note: β2M, beta-2 microglobulin; FISH, fluorescence in situ hybridization; Del, deletion; Tri, trisomy; Carcinoma of the breast (*N* = 6), melanoma (*N* = 2), carcinoma of the lung (*N* = 2), Acute myelocytic leukemia (N = 1), Carcinoma of prostate (N = 1), Carcinoma of stomach (*N* = 1).

**Table 2:** List of proteins in which the phosphorylation state was changed following exposure to exosomes secreted from CLL cells.

Number	Protein	Ratio E/C2	Number	Protein	Ratio E/C
**1**	sp|Q969G5|CAVN3_HUMAN	2075.64	**35**	sp|Q8NHW5|RLA0L_HUMAN	5.36
**2**	sp|P16989|YBOX3_HUMAN	657.79	**36**	sp|Q13283|G3BP1_HUMAN	5.35
**3**	sp|Q9BZE4|NOG1_HUMAN	192.53	**37**	sp|Q8NHW5|RLA0L_HUMAN	5.20
**4**	sp|O14950|ML12B_HUMAN	180.11	**38**	sp|P55081|MFAP1_HUMAN	5.11
**5**	sp|P35269|T2FA_HUMAN	176.30	**39**	sp|O60841|IF2P_HUMAN	4.98
**6**	sp|P62736|ACTA_HUMAN	101.09	**40**	sp|Q7L7L0|H2A3_HUMAN	4.60
**7**	sp|Q5SW79|CE170_HUMAN	97.33	**41**	sp|P51116|FXR2_HUMAN	4.20
**8**	sp|Q9NY61|AATF_HUMAN	88.28	**42**	sp|P42167|LAP2B_HUMAN	4.03
**9**	sp|P07355|ANXA2_HUMAN	57.33	**43**	sp|P13861|KAP2_HUMAN	3.92
**10**	sp|P19338|NUCL_HUMAN	44.40	**44**	sp|P17096|HMGA1_HUMAN	3.65
**11**	sp|Q6PD74|AAGAB_HUMAN	30.69	**45**	sp|Q15417|CNN3_HUMAN	3.50
**12**	sp|P58876|H2B1D_HUMAN	23.16	**46**	sp|P55010|IF5_HUMAN	3.29
**13**	sp|P48681|NEST_HUMAN	22.90	**47**	sp|P49959|MRE11_HUMAN	3.20
**14**	sp|Q6UN15|FIP1_HUMAN	19.60	**48**	sp|Q8IWX8|CHERP_HUMAN	2.97
**15**	sp|Q15154|PCM1_HUMAN	16.95	**49**	sp|Q9BW85|CCD94_HUMAN	2.95
**16**	sp|ALBU_BOVIN|	14.48	**50**	sp|Q6JBY9|CPZIP_HUMAN	2.89
**17**	sp|P46821|MAP1B_HUMAN	13.51	**51**	sp|Q9Y2W1|TR150_HUMAN	2.79
**18**	sp|P55081|MFAP1_HUMAN	11.36	**52**	sp|P05388|RLA0_HUMAN	2.73
**19**	sp|P46821|MAP1B_HUMAN	11.07	**53**	sp|O14950|ML12B_HUMAN	2.61
**20**	sp|P05386|RLA1_HUMAN	9.47	**54**	sp|P05388|RLA0_HUMAN	2.60
**21**	sp|Q8IWX8|CHERP_HUMAN	8.55	**55**	sp|P05388|RLA0_HUMAN	2.60
**22**	sp|P23497|SP100_HUMAN	8.31	**56**	sp|P11717|MPRI_HUMAN	2.57
**23**	sp|Q9NR30|DDX21_HUMAN	7.75	**57**	sp|P06748|NPM_HUMAN	2.43
**24**	sp|Q13242|SRSF9_HUMAN	7.69	**58**	sp|P63261|ACTG_HUMAN	2.36
**25**	sp|Q8NHW5|RLA0L_HUMAN	6.41	**59**	sp|P05386|RLA1_HUMAN	2.29
**26**	sp|Q9NYV4|CDK12_HUMAN	6.27	**60**	sp|Q15185|TEBP_HUMAN	2.22
**27**	sp|P29590|PML_HUMAN	6.05	**61**	sp|Q14676|MDC1_HUMAN	2.19
**28**	sp|Q9P2I0|CPSF2_HUMAN	5.97	**62**	sp|Q9NYF8|BCLF1_HUMAN	2.12
**29**	sp|Q96B36|AKTS1_HUMAN	5.84	**63**	sp|P48681|NEST_HUMAN	2.10
**30**	sp|ALBU_BOVIN|	5.81	**64**	sp|P16949|STMN1_HUMAN	2.07
**31**	sp|P55081|MFAP1_HUMAN	5.57	**65**	sp|P20290|BTF3_HUMAN	2.07
**32**	sp|P55081|MFAP1_HUMAN	5.54	**66**	sp|Q15185|TEBP_HUMAN	2.06
**33**	sp|Q15154|PCM1_HUMAN	5.40	**67**	sp|P05388|RLA0_HUMAN	2.05
**34**	sp|P35269|T2FA_HUMAN	5.37			

Yellow marks the proteins used for validation of the phosphoproteomic profiling are highlighted in yellow.

## Data Availability

Not relevant to the current study.

## Abbreviations

The following abbreviations are used in this manuscript:

CLL	Chronic lymphocytic leukemia
EVs	Extracellular vesicles

## References

[1] Paggetti J., Haderk F., Seiffert M., Janji B., Distler U., Ammerlaan W., Kim Y.J., Adam J., Lichter P., Solary E. (2015). Exosomes Released by Chronic Lymphocytic Leukemia Cells Induce the Transition of Stromal Cells into Cancer-Associated Fibroblasts. Blood.

[2] Burger J.A. (2011). Nurture versus Nature: The Microenvironment in Chronic Lymphocytic Leukemia. Hematol. Am. Soc. Hematol. Educ. Program.

[3] Burger J.A. (2013). The CLL Cell Microenvironment. Adv. Exp. Med. Biol..

[4] Lagneaux L., Delforge A., Bron D., De Bruyn C., Stryckmans P. (1998). Chronic Lymphocytic Leukemic B Cells but Not Normal B Cells Are Rescued from Apoptosis by Contact with Normal Bone Marrow Stromal Cells. Blood.

[5] ten Hacken E., Burger J.A. (2014). Molecular Pathways: Targeting the Microenvironment in Chronic Lymphocytic Leukemia—Focus on the B-Cell Receptor. Clin. Cancer Res..

[6] Rozovski U., Harris D.M., Li P., Liu Z., Wu J.Y., Grgurevic S., Faderl S., Ferrajoli A., Wierda W.G., Martinez M. (2016). At High Levels, Constitutively Activated STAT3 Induces Apoptosis of Chronic Lymphocytic Leukemia Cells. J. Immunol..

[7] Yeh Y.-Y., Ozer H.G., Lehman A.M., Maddocks K., Yu L., Johnson A.J., Byrd J.C. (2015). Characterization of CLL Exosomes Reveals a Distinct microRNA Signature and Enhanced Secretion by Activation of BCR Signaling. Blood.

[8] Syn N., Wang L., Sethi G., Thiery J.-P., Goh B.-C. (2016). Exosome-Mediated Metastasis: From Epithelial–Mesenchymal Transition to Escape from Immunosurveillance. Trends Pharmacol. Sci..

[9] Whiteside T.L. (2016). Tumor-Derived Exosomes and Their Role in Cancer Progression. Adv. Clin. Chem..

[10] Kumar B., Garcia M., Murakami J.L., Chen C.-C. (2016). Exosome-Mediated Microenvironment Dysregulation in Leukemia. Biochim. Biophys. Acta.

[11] Paggetti G., Leff D.R., Orihuela-Espina F., Mylonas G., Darzi A., Yang G.-Z., Menegaz G. (2015). The Role of the Posterior Parietal Cortex in Stereopsis and Hand–Eye Coordination during Motor Task Behaviours. Cogn. Process..

[12] Crompot E., Van Damme M., Pieters K., Vermeersch M., Perez-Morga D., Mineur P., Maerevoet M., Meuleman N., Bron D., Lagneaux L. (2017). Extracellular Vesicles of Bone Marrow Stromal Cells Rescue Chronic Lymphocytic Leukemia B Cells from Apoptosis, Enhance Their Migration and Induce Gene Expression Modifications. Haematologica.

[13] Lipshtein L. (2018). The Gray Faculty of Medical and Health Sciences. Master’s Thesis.

[14] Polk A., Lu Y., Wang T., Seymour E., Bailey N.G., Singer J.W., Boonstra P.S., Lim M.S., Malek S., Wilcox R.A. (2016). Colony-Stimulating Factor-1 Receptor Is Required for Nurse-Like Cell Survival in Chronic Lymphocytic Leukemia. Clin. Cancer Res..

[15] Pilling D., Fan T., Huang D., Kaul B., Gomer R.H. (2009). Identification of Markers That Distinguish Monocyte-Derived Fibrocytes from Monocytes, Macrophages, and Fibroblasts. PLoS ONE.

[16] Quan T.E., Cowper S.E., Bucala R. (2006). The Role of Circulating Fibrocytes in Fibrosis. Curr. Rheumatol. Rep..

[17] Hanna B.S., McClanahan F., Yazdanparast H., Zaborsky N., Kalter V., Rößner P.M., Benner A., Dürr C., Egle A., Gribben J.G. (2016). Depletion of CLL-Associated Patrolling Monocytes and Macrophages Controls Disease Development and Repairs Immune Dysfunction In Vivo. Leukemia.

[18] Berman J., Stoner G., Dawe C., Rice J., Kingsbury E. (1978). Histochemical Demonstration of Collagen Fibers in Ascorbic-Acid-Fed Cell Cultures. In Vitro.

[19] Kuter D.J., Bain B., Mufti G., Bagg A., Hasserjian R.P. (2007). Bone Marrow Fibrosis: Pathophysiology and Clinical Significance of Increased Bone Marrow Stromal Fibres. Br. J. Haematol..

[20] Mesaros O., Jimbu L., Neaga A., Popescu C., Berceanu I., Tomuleasa C., Fetica B., Zdrenghea M. (2020). Macrophage Polarization in Chronic Lymphocytic Leukemia: Nurse-Like Cells Are the Caretakers of Leukemic Cells. Biomedicines.

[21] Uziel O., Lipshtein L., Sarsor Z., Beery E., Bogen S., Lahav M., Regev A., Kliminski V., Sharan R., Gervits A. (2024). Chronic Lymphocytic Leukemia (CLL)-Derived Extracellular Vesicles Educate Endothelial Cells to Become IL-6-Producing, CLL-Supportive Cells. Biomedicines.

[22] Hetta H.F., Salama A., Aljuaid T.A., Mojmami Y.T., Alotibi R.S., Alqabaly A.M., Aldosari N.A., Alshahri S.A., Asiri W.I.A., Alamri R.S. (2026). Is Brukinsa (Zanubrutinib) a Safer Bruton’s Tyrosine Kinase (BTK) Inhibitor in Relapsed or Refractory Chronic Lymphocytic Leukemia? A Systematic Review and Meta-Analysis. Pharmaceuticals.

[23] Alatawi Y., Alanazi F.E., Alattar A., Alshaman R., Ramadan Y.N., Sayad R., Hetta H.F. (2025). Therapeutic Impact of Zanubrutinib in Chronic Lymphocytic Leukemia: Evidence from a Systematic Review and Single-Arm Meta-Analysis. Pharmaceuticals.

[24] Domagala M., Laplagne C., Leveque E., Laurent C., Fournié J.J., Espinosa E., Poupot M. (2021). Cancer Cells Resistance Shaping by Tumor Infiltrating Myeloid Cells. Cancers.

[25] Gutkin A., Uziel O., Beery E., Nordenberg J., Pinchasi M., Goldvaser H., Henick S., Goldberg M., Lahav M. (2016). Tumor Cells Derived Exosomes Contain hTERT mRNA and Transform Nonmalignant Fibroblasts into Telomerase Positive Cells. Oncotarget.

[26] Pang K., Wang W., Qin J., Shi Z., Hao L., Ma Y., Xu H., Wu Z., Pan D., Chen Z. (2022). Role of Protein Phosphorylation in Cell Signaling, Disease, and the Intervention Therapy. MedComm.

[27] Fonseca P., Vardaki I., Occhionero A., Panaretakis T. (2016). Metabolic and Signaling Functions of Cancer Cell-Derived Extracellular Vesicles. Int. Rev. Cell Mol. Biol..

[28] Rossignol M., Keriel A., Staub A., Egly J.M. (1999). Kinase activity and phosphorylation of the largest subunit of TFIIF transcription factor. J. Biol. Chem..

